# Endometrial sampling in low-risk patients with abnormal uterine bleeding: a systematic review and meta-synthesis

**DOI:** 10.1186/s12875-018-0817-3

**Published:** 2018-07-30

**Authors:** Brenda F. Narice, Brigitte Delaney, Jon M. Dickson

**Affiliations:** 1 Clinical Research Fellow in Obstetrics & Gynaecology; Academic Unit of Reproductive and Developmental Unit, University of Sheffield, Sheffield, S10 2SF UK; 2 Academic Unit of Primary Medical Care, University of Sheffield, Sheffield, S5 7AU UK

**Keywords:** Pipelle, Endometrial sampling, Abnormal uterine bleeding, Endometrial cancer, Endometrial hyperplasia, Premenopausal, Perimenopausal, Dilation and curettage

## Abstract

**Background:**

One million women per year seek medical advice for abnormal uterine bleeding (AUB) in the United Kingdom. Many low-risk patients who could be managed exclusively in primary care are referred to hospital based gynaecology services. Performing endometrial sampling (ES) in the community may improve care, reduce the rate of referrals and minimise costs. We aimed to search and synthesise the literature on the effectiveness of ES (Pipelle versus other devices) in managing AUB in low-risk patients.

**Methods:**

We undertook an electronic literature search in MEDLINE via OvidSP, Scopus, and Web of Science for relevant English-language articles from 1984 to 2016 using a combination of MeSH and keywords. Two reviewers independently pre-selected 317 articles and agreed on 60 articles reporting data from over 7300 patients. Five themes were identified: sample adequacy, test performance, pain and discomfort, cost-effectiveness, and barriers and complications of office ES.

**Results:**

Pipelle seems to perform as well as dilation and curettage and, as well or better than other ES devices in terms of sampling adequacy and sensitivity. It also seems to be better regarding pain/discomfort and costs. However, Pipelle can disrupt the sonographic appearance of the endometrium and may be limited by cervical stenosis, pelvic organ prolapse and endometrial atrophy.

**Conclusions:**

The current evidence supports the use of Pipelle in the management of low-risk women presenting in the outpatient setting with symptomatic AUB when combined with clinical assessment and ultrasound scanning. However, the implications of its widespread use in primary care are uncertain and more research is required.

**Electronic supplementary material:**

The online version of this article (10.1186/s12875-018-0817-3) contains supplementary material, which is available to authorized users.

## Background

Abnormal uterine bleeding (AUB), traditionally defined as uterine bleeding that is abnormal in volume, regularity, and/or timing [1] is common and affects 14–25% of women of reproductive age [2–4]. In the UK, approximately 1 million women seek medical advice for AUB every year, mostly in general practice [5, 6] and even though most cases could potentially be managed exclusively in primary care [7, 8], AUB is the fourth most common reason for referral to UK gynaecological services [6, 9, 10]. AUB has a major impact on quality of life [7], leads to 3.5 million days of work absence [11], and generates significant health care costs. Hospital referrals and hysterectomies are the major components of the £65 million/year treatment costs for AUB [10].

Most cases of AUB are benign and amenable to office-based treatments [12, 13]. However, patients often present with a myriad of symptoms, and their assessment requires training and expertise [13, 14]. The causes of AUB can be summarised using the PALM-COEIN acronym: polyps, adenomyosis, leiomyoma/fibroids, malignancy (and hyperplasia), coagulopathy, ovulatory disorders, endometrial, iatrogenic, and not otherwise classified [1].

Some patients who present with AUB will have endometrial hyperplasia or cancer which is the commonest gynaecological malignancy in the Western world. Even though the incidence rises after menopause, it can occur at all ages and 7% of cases are under 50 [15, 16]. This percentage seems to be rising with increasing prevalence of obesity and diabetes [17, 18].

In the UK, women with AUB who are deemed at high risk of endometrial cancer such as those with postmenopausal bleeding (PMB) or family history of gynaecological neoplasms, should be referred to secondary care [19]. For low-risk premenopausal women the guidance is not as clear. Although urgent referral is not required [20], national guidelines recommend that endometrial sampling (ES) should be performed in women over 40–45 years to exclude cancer [21, 22], but they do not specify whether ES should be performed in primary or secondary care [22].

In the UK, ES for AUB patients has not been traditionally undertaken in primary care. For many years, the standard management was dilation and curettage (D&C) in hospital under general anaesthesia [23–25]. However, the need for admission and the risks of perforation and haemorrhage made D&C unpopular [23, 25] and various ES devices were developed such as the Novak (a silastic cannula with a bevelled lateral opening [26]), the Tis-u-Trap (a plastic curette with suction [27]), the Vabra Aspirator (a stainless steel cannula connected to a vacuum pump [28]), the Endorette (a plastic cannula with multiple openings [29]), the Tao Brush (a sheath brush device [30]), the Cytospat (a polypropylene cannula with a rhomboid head [31]), the Accurette (a quadrilateral-shaped curette with four cutting edges [32]) and the Pipelle, the most widely used device in the UK (a flexible plastic tube with a distal circular port [27]).

We conducted a systematic review of the literature to identify existing evidence about the effectiveness of Pipelle compared with other ES techniques for assessing low-risk women with AUB which could inform the development of new care pathways in primary care.

### Why this study was necessary

Endometrial sampling is thought to be a safe and effective method for histological assessment of the endometrium. It is used as an alternative to the more invasive method of D&C. This is the first review to focus on AUB in low-risk pre- and perimenopausal women. We conclude that ES is a valuable tool in the assessment of these patients and that Pipelle is the best outpatient device available. The evidence supports the use of Pipelle in the outpatient setting but more research is required to assess its impact if introduced as routine management of AUB in the community.

## Methods

### Literature search

We used the PICO approach to develop a systematic search strategy [33]. We searched MEDLINE via OvidSP, Scopus, and Web of Science. For Medline, key concepts were identified (endometrial hyperplasia/cancer, abnormal uterine bleeding, endometrial sampling), a list of synonyms was generated for each concept and these lists were used to identify MeSH terms for the search (Additional file 1). Similar search strategies were used for Scopus and Web of Science (Additional file 1), always limited to papers from 1984 (when Pipelle was first introduced [34]) to 2016, written in English and involving humans.

We included papers investigating ES in women with AUB. We also considered studies in patients with known cancer; although these studies do not inform the indication of ES in primary care, they were an important source to evaluate test performance. We included review articles and opinion pieces. We excluded papers exclusively analysing postmenopausal patients, papers where the indication was assessment of fertility or recurrent miscarriage and papers where ES was assisted by hysteroscopy (unless this was used as a comparator to blind ES).

**Fig. 1:**
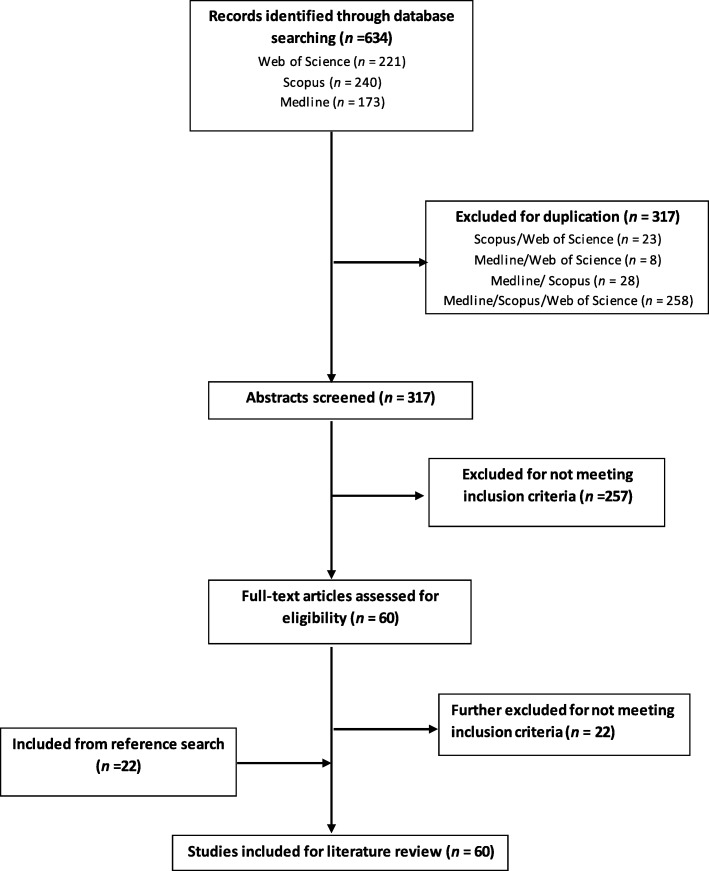
PRISMA flow diagram for study selection

The initial search generated 173 results for Medline, 240 for Scopus, and 221 for Web of Science, totalling 634 search hits across all databases, 317 of which were excluded for duplication. The remaining 317 articles were assessed for inclusion using the titles and abstracts. The assessment was independently repeated by a second reviewer and a consensus was reached. After this process, 257 papers were excluded and the full text of 60 papers were read. Twenty-two further papers were excluded while another 22 papers were added from reference search, giving a final list of 60 papers. This selection included 16 randomized controlled trials (RCT), 26 prospective studies, 6 retrospective studies, 5 reviews, 2 meta-analyses, 1 survey, and 4 brief communications and letters to the editor, which were included in the final analysis providing data over 7300 women (Fig. 1).

#### Bias risk assessment

The quality of the RCTs was assessed using the standard Cochrane Risk of Bias tool [35], and the quality of observational studies was analysed with the modified Agency for Healthcare Research and Quality (AHRQ) quality assessment criteria [36].

## Results

### Risk of Bias / quality of studies

The overall quality of the RCTs was poor (*n* = 4) to moderate (*n* = 12), no high quality studies were identified. For observational studies, the risk of bias ranged from 31 to 79% with a mean weighted score 52.8% SD ± 11.8% which again suggests overall moderate quality [37]. See additional online content for tabulated assessments of individual studies (Additional file 2: TableS1 and 2, Additional file 2).

### Five themes

We identified five major themes in the literature: (1) sample adequacy (defined as enough tissue to be analysed by pathologists [38]); (2) test performance when compared with hysterectomy and D&C; (3) acceptability by the patient in terms of pain experienced during sampling; (4) the costs of taking outpatient endometrial biopsies; and (5) the barriers and complications of performing office ES.

All studies, except for one, were carried out in specialised outpatient gynaecology clinics or hospital services (secondary care) [39]. Only one study looked exclusively at premenopausal women [40]. The rest reported on cohorts of both pre- and post-menopausal women or they did not present results based on menopausal status. Most studies included women with symptomatic AUB and no risk of endometrial carcinoma. However, five studies targeted women with endometrial cancer to correlate pre-operative Pipelle with the hysterectomy histopathology [41–45]. Studies are summarised in Table 1.

**Table 1:** Comparison of the RCTs, prospective and retrospective studies included in this literature review. Papers have been grouped by intervention/ comparator

Study	Type of study	Age of participants (mean ± SD)	Intervention (n) vs Comparator (n)	Outcome	Pain	Cost
Pipelle versus D&C +/− Hysterectomy						
[23] Rauf et al. Pakistan 2014	RCT	46.3 ± 4.45	Pipelle (102) vs D&C (101)	Adequacy Pipelle 98% D&C 100%	Pipelle less painful	Pipelle cheaper
[46] Liu et al. China 2015	Prospective Sequential	43.6	Pipelle vs D&C (245)	Adequacy Pipelle 91.02% D&C 92.24%	Pipelle less painful	N/A
[47] Gungorduk et al. Turkey 2013	Prospective	Pipelle: 49.8 ± 6.1 D&C: 48.2 ± 6.5	Pipelle + hysterectomy (78) vs D&C + hysterectomy (189)	Adequacy Pipelle 95% D&C 96% Concordance Pipelle + hysterectomy 62% D&C + hysterectomy 67%	Pipelle less painful	Pipelle cheaper
[48] Kazandi et al. Turkey 2012	Prospective Sequential	48 ± 9.43	Pipelle + hysterectomy Vs D&C + hysterectomy (66)	Adequacy Pipelle 93% D&C 96% Concordance Pipelle and D&C 66% Pipelle & hysterectomy 64%	Pipelle less painful	Pipelle cheaper
[49] Demirkiran et al. Turkey 2012	Prospective	45.3	Pipelle + hysterectomy (212) vs D&C + hysterectomy (161)	Adequacy Pipelle 97% D&C98% Concordance Pipelle and D&C 84% Pipelle & hysterectomy 67% D&C and hysterectomy 80%	Pipelle less painful	Pipelle cheaper
[43] Sany et al. UK 2011	Retrospective	?	Pipelle + hysterectomy vs D&C + hysterectomy (total 191)	Concordance Both techniques 78%	N/A	N/A
[45] Daud et al. UK 2011	Retrospective	55.7 ± 11.4	Pipelle ± hysterectomy (75) vs D&C ± hysterectomy (220)	Concordance Pipelle + hysterectomy 76% D&C + hysterectomy 86%	N/A	N/A
[24] Fakhar et al. Pakistan 2008	Prospective Sequential	45.4 ± 7.2	Pipelle versus (D&C) (100)	Adequacy Pipelle 98% D&C 100% NPV for endometrial carcinoma Pipelle 100%	N/A (both techniques under GA)	Pipelle cheaper
[44] Huang et al. USA 2006	Retrospective + Letter	?	Pipelle + hysterectomy (253) vs D&C + hysterectomy (93)	Concordance Pipelle and hysterectomy 93.8% (low grade cancer) & 99.2% (high grade cancer) D&C and hysterectomy 97% (low grade cancer) & 100% (high grade cancer)	N/A	N/A
[37] Macones et al. 2006						
[66] Machado et al. Spain 2002	Retrospective	Post-menopausal (68) Pre- or peri-menopausal (100)	Pipelle (168) vs D&C (92) ± Hysterectomy (76)	Accuracy Sensitivity 84.2% Specificity 99.1%	N/A	N/A
[51] Kavak et al. Turkey 1996	Prospective	50.8 ± 7.8	Pipelle ± TVS (78) vs D&C (78)	Concordance Sensitivity: 73% (increased to 90% with TVS) Specificity: 100%	N/A	N/A
[50] Ben-Baruch et al. Israel 1993	Prospective	Pre- and post-menopausal	Pipelle (172) vs D&C (97)	Adequacy Pipelle 90.6% D&C 68%	N/A	N/A
[68] Sanam et al. Iran 2015	Prospective	> 35	Pipelle (130) vs D&C (130)	Concordance Pipelle and D&C 94% Adequacy Pipelle 84.6% D&C 90%	N/A	Pipelle cheaper
[75] Gordon New Zealand 1999	Prospective	47.2 ± 1.8	Pipelle (100) vs D&C or hysterectomy (n =?)	Adequacy Pipelle 67%	N/A	N/A
[69] Goldchmit et al. Israel 1993	Prospective Sequential	48.1	Pipelle and TVS vs D&C (176)	Concordance Pipelle & D&C 90% (increased to 92% with TVS)	N/A	N/A
[52] Abdelazim et al. Turkey 2013	Prospective Sequential	44.5	Pipelle vs D&C (143)	Adequacy Pipelle 97.9% D&C 100% NPV for endometrial polyp Pipelle 89.6%	N/A	N/A
[72] Shams Pakistan 2012	Prospective Sequential	47.94	Pipelle vs D&C (50)	N/A	Pipelle less painful	Pipelle cheaper
[53] Rezk et al. Egypt 2016	Prospective	Pipelle: 47.2 ± 3.8 D&C: 46.9 ± 4.1	Pipelle (270) vs D&C (268)	Adequacy No difference (p˃0.05)	D&C less painful	N/A
Pipelle versus Vabra +/− Hysterectomy						
[54] Eddowes et al. UK 1990	Prospective Sequential	41.6	Pipelle vs Vabra Aspirator (100)	Adequacy Pipelle 88% Vabra Aspirator 88%	Pipelle less painful	Pipelle cheaper
[55] Naim et al. Malaysia 2007	RCT	> 45	Pipelle (76) vs Vabra Aspirator (71)	Adequacy Pipelle 73.3% Vabra 52.4%	N/A	Pipellle cheaper
[28] Kaunitz et al. USA 1988	Prospective Sequential	46	Pipelle vs Vabra (56)	Adequacy Pipelle & Vabra 91% Concordance Pipelle & Vabra 89%	Pipelle less painful	Pipelle cheaper
[56] Rodriguez et al. USA 1993	RCT	?	Pipelle (12) vs Vabra (13) vs Hysterectomy (25)	Surface being sampled: Pipelle 4.2% Vabra 41.6%	N/A	N/A
Pipelle versus Tao Brush+/− Hysteroscopy						
[30] Williams et al. UK 2008	RCT Sequential	Moderate risk: 45.2 (SE 0.26)	For moderate risk Pipelle (34) Tao Brush (29)	Adequacy Both techniques 84% No significant difference for premenopausal	Tao Brush less painful	N/A
[57] Critchley et al. UK 2004	RCT	Moderate risk: pre-menopausal ˃40 or < 40 with risk for endometrial cancer Low risk	Pipelle vs Tao Brush Moderate risk (Total 326) Low risk (Total 157) ± hysteroscopy ± TVS	Successful completion of investigation: Pipelle 85% Adequacy of sample with Pipelle: Moderate risk 79%	Tao Brush less painful than Pipelle	Minimal difference
[58] Yang et al. USA 2003	Prospective Sequential	24–86	Pipelle (79) vs Tao Brush (79)	Factors affecting sensitivity: tumour size, type, location within the uterus, sampling mechanism and preparation method	N/A	N/A
[59] Del Priore et al. USA 2001	RCT Sequential	Pre-menopausal: 46 Post-menopausal: 61	Tao Brush vs Pipelle (50)	Sensitivity: Pipelle 86% Tao Brush 95.5% Specificity: Both 100%	N/A	Tao Brush cheaper than D&C
[60] Yang et al. USA 2000	Prospective Sequential	58	Tao Brush vs Tao Brush + Pipelle (25)	Adequacy Tao Brush 98% Pipelle 88%	Tao Brush less painful	Comparable
Pipelle versus Novak						
[40] Henig et al. USA 1989	RCT	Pre-menopausal	Pipelle (50) Vs Novak (50)	Adequacy Pipelle 94% Novak 98%	Better tolerance with Pipelle	N/A
[26] Stovall et al. USA 1991	RCT	Pipelle: 40 Novak: 44	Pipelle (149) vs Novak (126)	Adequacy Pipelle 87.2% Novak 90.5%	Pipelle less painful	Novak might be cheaper
[61] Silver et al. USA 1991	RCT Sequential	28–76	1st Pipelle then Novak (26) vs 1st Novak then Pipelle (29)	Adequacy Similar	Pipelle less painful	N/A
Pipelle versus Hysterectomy						
[67] Guido et al. USA 1995	Prospective Sequential	61	Pipelle vs Hysterectomy (71)	Adequacy Pipelle 97% Concordance Pipelle & hysterectomy 83%	N/A	N/A
[42] Ferry et al. UK 1993	Prospective Sequential	?	Pipelle vs Hysterectomy (37)	Concordance Pipelle & hysterectomy 67%	N/A	N/A
[41] G Zorlu et al. Turkey 1994	Prospective Sequential	51	Pipelle vs Hysterectomy (26)	Concordance Pipelle & hysterectomy 95%	Mild pain and discomfort with Pipelle	N/A
Pipelle versus Explora +/− Accurette						
[62] Leclair et al. USA 2011	RCT	Pipelle: 45.2 ± 7.3 Explora: 46.1 ± 7.7	Pipelle (37) vs Explora (32)	Adequacy Pipelle 91% Explora 97%	No differences seen	N/A
[32] Lipscomb et al. USA 1994	RCT	N/A Pre- and post-menopausal	Pipelle (85) vs Accurette (81) vs Explora (82)	Adequacy Pipelle 85.2% Accurette 72.5% Explora 85.4%	No significant difference in pain score	N/A
Pipelle versus Infant Feeding Tube (IFT)						
[63] Bhide et al. UK 2007	Prospective	?	Pipelle (29) vs IFT (31)	Adequacy Pipelle 73% IFT 71%	Less pain with IFT	N/A
Pipelle Mark 2 versus Pipelle Mark 2 + hysteroscopy						
[71] Polena et al. France 2006	Prospective Sequential	50	Pipelle Mark 2 vs Pipelle Mark 2 ± hysteroscopy (97)	Adequacy of Pipelle Mark 2 88.7%	No difference with conventional Pipelle	Slightly more expensive than conventional Pipelle
Pipelle versus Tis-u-Trap						
[27] Koonings et al. USA 1990	RCT	Pipelle: 42.9 Tis-u-trap: 42.3	Pipelle + hysterectomy (74) vs Tis-u-trap + hysterectomy (75)	Adequacy Pipelle 87.8% Tis-u-trap 84% Concordance Pipelle & hysterectomy 85% Tis-u-trap & hysterectomy 92%	N/A	Pipelle cheaper
Pipelle versus Endorette						
[29] Moberger et al. Sweden 1998	RCT Sequential	57.5 ± 11.5	Pipelle vs Endorette (152)	Adequacy and concordance No difference	No significant difference	N/A
Pipelle versus Cytospat +/− Hysterectomy						
[31] Antoni et al. Spain 1996	RCT	48.6 ± 9	Pipelle ± hysterectomy or D&C (191) vs Cytospat ± hysterectomy or D&C (174)	Adequacy Pipelle 75% Cytospat 76% Concordance Pipelle: Benign 84%, Hyperplasia 71%, Malignancy 60% Cytospat: Benign 82%, Hyperplasia 60%, Malignancy 60%	Better tolerance for Pipelle	Pipelle cheaper
Pipelle versus D&C +/− Hysteroscopy +/− TV US						
[85] Tahir et al. UK 1999	RCT	35	Inpatient: Hysteroscopy & D&C (200) vs Outpatient: Pipelle +/− TV US +/− Hysteroscpy (200)	Adequacy No difference Concordance Inpatient: 100& Outpatient: 82&	More pain in outpatient	N/A
Others						
[73] Trolice et al. USA 2000	RCT Anaesthesia for Pipelle	Lidocaine: 42.1 ± 11.9/ Saline: 44.9 ± 12.5	Lidocaine (19) vs Saline (22)	Significant reduction of pain with lidocaine	Less pain with intervention	N/A
[34] Cornier France 1984	Brief communication	Mostly pre-menopausal	Pipelle (250) No control	Useful for histologic dating of the endometrium	Little discomfort	Low cost
[74] Frishman USA 1990	Letter in response to study [27]	N/A	Pipelle versus Tis-u-Trap	N/A	N/A	Pipelle cheaper
[38] Mc Cluggage Northern Ireland 2006	Review	N/A	Pipelle versus other ES	Difficulties of processing outpatient ES samples	N/A	N/A
[79] Van Den Bosch Belgium 2005	Prospective sequential	Pre-menopausal: 41.6 ± 8.7 Post-menopausal: 59 ± 9.9	US before and after Pipelle (99)	Thickness of the endometrium ET on average 0.4 mm less after performing Pipelle	N/A	N/A
[76] Brandner et al. Germany 2000	Review	N/A	N/A	Progression of endometrial lesions (potential limitations for ES)	N/A	N/A
[80] Dijkhuizen et al. The Netherlands 2000	Meta-analysis	39 studies including 7914 patients	Different ES	Pipelle is superior to other ES for diagnosing cancer/ hyperplasia	N/A	N/A
[25] Cooper et al. USA 2000	Review	N/A	N/A	Directed biopsy with Hysteroscopy: most accurate ES (not for primary care)	N/A	N/A
[14] Farquhar et al. New Zealand 1996	Survey	68 replies from O&G consultants (48% of all contestants)	N/A	Management of menorrhagia in primary care	N/A	N/A
[78] Youssif et al. Australia 1995	Review	N/A	N/A	Effectiveness and safety of Pipelle	N/A	N/A
[77] Dantas et al. Brazil 1994	Letter	Nurses vs doctors performing Pipelle	N/A	Adequacy No difference	N/A	N/A
[82] Clark et al. UK 2002	Systematic review and meta-analysis	Mixed pre- (21%) and pos-tmenopausal (79%)	Pipelle vs other outpatient techniques	Likelihood ratio of endometrial cancer when Pipelle is: -ve: 0.1 +ve: 64.6	N/A	N/A
[86] Ahonkallio et al. Finland 2009	Prospective	Range 47–52 Post ablation	Pipelle (57)	Adequacy 29% failure If endometrium < 5 mm 5% failure if endometrium > 5 mm	N/A	N/A
[81] Du et al. China 2016	Review	N/A	N/A	Most appropriate ES devices for endometrial lesions	Little discomfort	N/A
[64] Masood et al. Pakistan 2015	Cross sectional	Pre- and post-menopausal 35–48	Pipelle (126) vs no comparator	Adequacy Pipelle 96.82%	N/A	Cost-effective
[39] Seamark UK 1998	Prospective	≥40 42–74 Primary care population	Pipelle (38) vs no comparator	Adequacy Pipelle 76%	N/A	N/A
[70]Seto UK 2016	Retrospective	Pre-menopausal 46.1 ± 4.6 Post-menopausal 57.2 ± 8.1	Pipelle against hysteroscopy	Positive predictive value for endometrial polyp Pipelle (pre-menopausal) 53.7%	N/A	N/A
[65] Piatek et al. Poland 2016	Retrospective	Pre- and post-menopausal	Pipelle (312) vs no comparator	Adequacy 83.01%	N/A	N/A

*ES* Endometrial sampling, *AUB* Abnormal uterine bleeding, *RCT* Randomized controlled trials, *US* Transvaginal ultrasound, *N/A* Non-applicable,? Unknown

### Sample adequacy

Overall, the literature showed that the adequacy of material retrieved for histological analysis with Pipelle was comparable to D&C and superior to most of the other devices in pre-menopausal women. Ten studies [23, 24, 46–53] assessed Pipelle against D&C in premenopausal women, reporting rates of adequacy ranging from 89.74% [51] to 98% [23, 24] (Table 1).

Three studies compared the sample adequacy of Pipelle and Vabra Aspirator [28, 54, 55]. One of these studies [55] showed better rates for Pipelle (73.3% versus 52.4%, *P =* 0.02) whereas the remaining two could not identify any significant difference between both techniques (one study reported 91% for both techniques [28] whereas the other showed 89.79% for Vabra versus 88% for Pipelle [54], no *P* values provided) (Table 1). We also found a RCT which reported that Pipelle despite being equal or superior to Vabra in terms of sample adequacy only assesses 4.2% of the endometrium versus 41.6% with Vabra [56].

Five studies including mixed cohorts of pre- and postmenopausal women compared sample adequacy between Pipelle and Tao Brush [30, 57–60]. Despite one study suggesting that Tao Brush bendable wire should improve sampling of the uterine lateral walls when compared to Pipelle more rigid structure, none of the studies showed significant differences in premenopausal populations [58] (Table 1).

Two studies [40, 61] also compared Pipelle to Novak and found no statistically significant difference in terms of adequacy of sample, which varied from 83 to 94% for Pipelle and from 85 to 98% for Novak [40, 61] (Table 1). Six additional studies did not find a significant difference when comparing Pipelle with other less popular ES techniques such as Explora [32, 62] (85.4–97% for Explora versus 85.2–91% for Pipelle), Tis-u-trap [27] (88% for Pipelle versus 84% for Tis-u-Trap *P =* 0.5), Endorette [29] (56% for Endorette versus 43% for Pipelle), infant feeding tube [63] (73% for Pipelle versus 71% for IFT) and Cytospat [31] (Pipelle 74.9% versus 75.9% for Cytospat).

Three studies [39, 64, 65] assessed the ability of Pipelle to retrieve enough tissue for histological analysis without comparing it to other devices, and reported a success rate of 76% in GP practices [39], and a range from 83.01 to 96.82% in secondary care [64, 65] (Table 1).

### Test performance

Nine studies compared the histopathological diagnosis of pre-operative Pipelle and D&C with the final results from hysterectomy (the gold standard diagnostic technique for uterine disorders) [41, 43–45, 47–49, 66, 67]. For Pipelle, the sensitivity ranged from 62% [47] to 99.2% [44] and for D&C sensitivity varied from 67% [47] to 100% [44]. One of these studies applied Pipelle and D&C sequentially before hysterectomy [48], while the rest were multi-arm studies [41, 43–45, 47, 49, 66, 67] (Table 1).

At least 5 studies [43, 48, 49, 68, 69] also reported on the concordance between Pipelle and D&C with values that ranged from 66% [48] to 94% [68].

One retrospective study which compared Pipelle samples suggestive of endometrial polyps with subsequent hysteroscopically-guided polypectomies reported Pipelle had a positive predictive value of 55.3% for sampling polyps in premenopausal women [70]. Pipelle has also been reported to have 100% negative predictive value (NPV) for endometrial carcinoma and hyperplasia [24] and up to 99.2% NPV for endometritis and 89.6% for endometrial polyps [52] (Table 1).

### Pain / discomfort

Most studies included in this review performed ES on awake patients, but only 23 studies formally assessed patients’ pain using visual pain analogue scales and questionnaires (Table 1). A total of 15 studies reported that most patients experienced minimal discomfort with Pipelle [23, 26, 28, 31, 34, 40, 41, 46–49, 54, 61, 71, 72], three did not find any significant difference between Pipelle and Explora [32, 62] and Pipelle and Endorette [29], three concluded that Tao Brush was better tolerated than Pipelle [30, 57, 60] and one study showed less discomfort when using an infant feeding tube as a prototype [63]. A RCT also reported that paracervical lidocaine during Pipelle may decrease pain when compared to placebo [73] (Table 1).

### Costs of outpatient endometrial sampling

A total of 17 studies assessed the cost-effectiveness of Pipelle though none formally provided a health economic analysis [23, 24, 26–28, 31, 47–49, 54, 55, 57, 60, 68, 71, 72, 74]. Some of the factors they considered when assessing the total cost of ES were the need for general anaesthesia and hospital admission [23, 72] and the cost of operative hysteroscopy/ D&C following a failed office ES or an inadequate sample [55]. Fifteen studies showed Pipelle was cheaper than the alternative ES [23, 24, 26–28, 31, 47–49, 54, 55, 57, 68, 72, 74] and two did not find significant differences between Pipelle and Pipelle Mark 2 [71], and Pipelle and Tao Brush [60]. Two studies concluded that the Vabra was cheaper than Pipelle given its multiple use [26] but when all costs were considered including the need for follow-up for failed procedures, the average cost of Pipelle per patient was approximately 30% cheaper than the Vabra aspirator [55] (Table 1).

### Barriers and complications to endometrial sampling in primary care

Several limitations to successful ES were reported including cervical stenosis and pelvic organ prolapse which hindered the access to the uterine cavity [24, 75] as well as focal endometrial pathology (e.g. endometrial polyps and submucosal fibroids) and endometrial atrophy which reduces sample adequacy [30, 46, 69, 75, 76]. Lack of experience was also linked to inadequate sampling with higher failure rates seen in registrars (39%) than in consultants (25%), (*P* = 0.13)) [75]. However, a study which compared sample adequacy between nurses (83.3%) and doctors (80%), *P* > 0.05, concluded that with the right training the ability to perform successful Pipelle is independent of professional category [77].

While few complications have been associated with Pipelle [73]. [78], mainly discomfort and false negative results, a study showed that Pipelle makes the endometrium approximately 0.4 mm thinner and creates echogenic spots which can be misinterpreted as sonographic lesions if the ultrasound is not performed prior to ES [79] (Table 1).

## Discussion

Our aim was to search and synthesise the whole range of literature on ES in AUB in low-risk patients to guide further research and develop new evidence-based care pathways in primary care. Overall, the evidence that we have identified supports the use of ES in the outpatient setting and is a valuable source for the development of new care pathways in primary care.

To the best of our knowledge, this study is the first systematic review to primarily focus on the role of ES in assessing and managing AUB in low-risk women in the outpatient setting [25, 78, 80–82]. The available evidence shows that when Pipelle is combined with clinical assessment and ultrasound findings, it becomes a valuable tool for investigating AUB in low-risk women. Pipelle seems to perform as well or better than any other ES device in terms of sampling adequacy and sensitivity, with comparable results to D&C which for years was the standard technique for obtaining endometrial tissue in patients with AUB [78]. Furthermore, Pipelle seems to be cost-effective and better tolerated in terms of pain/discomfort [83]. However, its use has shown to be limited by cervical stenosis, pelvic organ prolapse and endometrial atrophy [24, 75]. Since Pipelle causes changes in the endometrium, it should not be performed before USS [79], and if the ultrasound reports localised lesions, a hospital referral for a hysteroscopy-guided biopsy may prove more useful than performing a blind Pipelle [84] given its limited sensitivity for focal lesions [47, 70].

Despite our robust and thorough literature search, we have noted some limitations in the available evidence. We only identified one study which was conducted on a primary care population by general practitioners [39] and one study which looked exclusively at premenopausal patients [40] and therefore, our conclusions are mainly based on studies which were carried out in either outpatient specialised clinics or hospital departments on a mixed cohort of pre- and postmenopausal women. Many of the studies that we identified were of poor or moderate methodological quality with wide-ranging inclusion and exclusion criteria (see Additional file 2). This heterogeneity may partly be responsible for the significant variability seen in terms of the sensitivity and specificity of Pipelle for detecting endometrial hyperplasia/cancer.

A meta-analysis was beyond the scope of this paper but critical appraisal and analysis of pooled data from diagnostic studies is an important next step in establishing the utility of ES. Given the limited information about the true test performance of ES in the community, it is not possible for clinicians to quantify the risk of hyperplasia/cancer (or other pathology) based only on ES. This is especially pertinent when the sample result is normal but the patient is still symptomatic; clinicians should then continue to consider the possibility of false negative results e.g. undiagnosed cancer/hyperplasia in these patients.

## Conclusions

The evidence we analysed suggests that performing ES in the outpatient setting may allow effective management of low-risk women with AUB in primary care without referral to a hospital. But the false negative rate, health economics and implications of such a change in practice are still unknown and more research is required.

## Additional files

Additional file 1: Literature search strategy. Search strategies and key words employed in the literature review. (DOCX 11 kb).

Additional file 2: Study quality assessment. Assessment of methodological quality of the studies included in the literature review. (DOCX 62 kb).

## Abbreviations

AUB: Abnormal uterine bleeding
D&C: Dilation and curettage
ES: Endometrial sampling
PMB: Postmenopausal bleeding
RCT: Randomised controlled trial
USS: Ultrasound scan
