# Supplementary material for: Endometrial sampling in low-risk patients with abnormal uterine bleeding: a systematic review and meta-synthesis
Source: BMC Fam Pract. 2018 Jul 30;19:135. doi: 10.1186/s12875-018-0817-3 (PMC6066914; doi:10.1186/s12875-018-0817-3)
Supplement: Supplementary file 1 — Literature search strategy. Search strategies and key words employed in the literature review. (DOCX 11 kb). [file 12875_2018_817_MOESM1_ESM.docx]

**Additional file 1**

**Search strategies**

**Medline/ Scopus/ Web of Science**

(“endometrial hyperplasia” OR “endometrial cancer” OR “endometrial tumour” OR “endometrial neoplasm”)

AND

(menorrhagia OR “dysfunctional uterine bleeding” OR “abnormal uterine bleeding” OR “uterine haemorrhage” OR “metrorrhagia”)

AND

(“endometrial sampling” OR “endometrial biopsy” OR Pipelle)

English/ human studies/ 1984-2016
