# Supplementary material for: Endometrial sampling in low-risk patients with abnormal uterine bleeding: a systematic review and meta-synthesis
Source: BMC Fam Pract. 2018 Jul 30;19:135. doi: 10.1186/s12875-018-0817-3 (PMC6066914; doi:10.1186/s12875-018-0817-3)
Supplement: Supplementary file 2 — Study quality assessment. Assessment of methodological quality of the studies included in the literature review. (DOCX 62 kb). [file 12875_2018_817_MOESM2_ESM.docx]

**Additional file 2**

Twelve RCTs concealed the allocation with opaque sealed envelopes or other methods [26,27,29,30,32,40,56,57,61,62,73,85].The method of allocation was unclear in the remaining four RCTs (Table 1). Blinding was assessed separately for patients, operators, and pathologists. Three studies blinded the procedure to patients [27,30,73] but only in one RCT, which assessed the role of topical anaesthetics pre-insertion of the Pipelle, were operators blinded [73].

Sixteen RCTs and prospective studies also formally reported blinding pathologists to the intervention [23,24,27–29,31,32,40,46,48,55,56,58,61,73,85]. Only one study specified using multiple pathologists to analyse samples in order to reduce observer bias [69], but they did not comment on whether these pathologists were blinded to the intervention.

Most studies had complete data sets and only a few formally reported on missing data [28,31,45,47,62,66,68]. Four studies specified an intention-to-treat analysis [30,50,57,61].

Many studies used D&C as a control instead of hysterectomy, considered the gold standard for assessing the endometrium, slightly increasing the risk of bias (Manuscript, Table 1).

All in all, most RCTs were classified as of moderate quality (Table 1) whereas the quality of prospective studies ranged from 31% to 79% (X= 52.8%, SD ± 11.8%) according to AHRQ modified criteria (Table 2).

| **Study** | **Random sequence operation** | **Allocation concealment** | **Blinding of participants and operators** | **Blinding of outcome assessment**  **(pathologists)** | **Incomplete outcome data** | **Selective outcome reporting** | **Overall quality** |
| --- | --- | --- | --- | --- | --- | --- | --- |
| [23] Rauf et al | Yes | No | No | Yes | No | No | Poor |
| [27] Koonings et al | Yes | Yes | Yes (participants) | Yes | No | No | Moderate |
| [40] Henig et al | Yes | Yes | No | Yes | No | No | Moderate |
| [62] Leclair et al | Yes | Yes | No | Unclear | Yes | Yes | Moderate |
| [30] Williams et al | Yes | Yes | Yes (participants) | No | Yes | Yes | Moderate |
| [55] Naim et al | Unclear | Unclear | No | Yes | Yes | Yes | Poor |
| [57] Critchley et al | Yes | Yes | No | No | Yes | Yes | Moderate |
| [59] Del Priore et al | Unclear | No | No | No | Unclear | Unclear | Poor |
| [73] Trolice et al | Yes | Yes | Yes (participants and operators) | Yes | No | No | Moderate |
| [85] Tahir et al | Yes | Yes | No | Yes | No | No | Moderate |
| [29]Moberger et al | Yes | Yes | No | Yes | No | No | Moderate |
| [31] Antoni et al | Yes | No | No | Yes | Yes | Yes | Poor |
| [32] Lipscomb et al | Yes | Yes | No | Yes | No | Yes | Moderate |
| [56] Rodriguez et al | Yes | Yes | No | Yes | No | No | Moderate |
| [26] Stovall et al | Yes | Yes | No | Unclear | No | No | Moderate |
| [61] Silver et al | Yes | Yes | No | Yes | No | No | Moderate |

***Table 1.*** Assessment of risk of bias in RCTs using the Cochrane Risk of Bias tool.

***Table 2.*** Assessment of risk of bias in observational studies using modified AHRQ quality assessment criteria.

| **Study** | **Study question**  **(%)** | **Study population + Comparability of subjects**  **(%)** | **Exposure or Intervention**  **(%)** | **Outcome measures**  **+ Statistical analysis**  **(%)** | **Results +**  **Discussion**  **+ Funding**  **(%)** | **Weighted score**  **(%)** |
| --- | --- | --- | --- | --- | --- | --- |
| [46] Liu et al | 2 | 2.5 + 6 | 9 | 15 + 12 | 6 + 4 + 5 | 61.5 |
| [47] Gungorduk et al | 2 | 5 + 21 | 9 | 15 + 10 | 6 + 3 + 0 | 71 |
| [48] Kazandi et al | 2 | 2.5 + 9 | 9 | 20 + 12 | 6 + 5 + 0 | 65.5 |
| [49] Demirkiran et al | 2 | 5 + 3 | 9 | 15 + 10 | 6 + 4 + 5 | 59 |
| [43] Sany et al | 1 | 3 + 10 | 9 | 15 + 0 | 3 + 2 + 0 | 43 |
| [45] Daud et al | 2 | 5 + 6 | 9 | 10 +10 | 6 + 5 + 5 | 58 |
| [24] Fakhar et al | 2 | 5 + 14 | 9 | 15 + 0 | 3 + 2 + 5 | 55 |
| [44] Huang et al | 2 | 5 + 3 | 9 | 10 + 5 | 3 + 3 + 0 | 40 |
| [66] Machado et al | 2 | 4 + 3 | 9 | 10 + 3 | 3 + 3 + 0 | 37 |
| [51] Kavak et al | 2 | 5 + 8 | 11 | 11 + 8 | 6 + 3 + 0 | 54 |
| [50] Ben-Baruch et al | 2 | 4 + 8 | 9 | 14 + 5 | 6 + 1 + 0 | 49 |
| [68] Sanam et al | 1 | 5 + 15 | 9 | 15 + 14 | 6 + 2 + 0 | 67 |
| [75] Gordon et al | 1 | 5 + 9 | 9 | 13 + 3 | 6 + 5 + 0 | 51 |
| [69] Goldchmit et al | 2 | 3 + 6 | 11 | 13 + 10 | 6 + 3 +0 | 54 |
| [54] Eddowes et al | 1 | 4 + 3 | 11 | 15 + 0 | 3 + 2 + 0 | 39 |
| [28] Kaunitz et al | 2 | 2 + 6 | 9 | 20 + 2 | 5 + 2 + 0 | 48 |
| [60] Yang et al (2000) | 2 | 5 + 9 | 9 | 13 + 12 | 6 + 4 + 4 | 64 |
| [58] Yang et al (2002) | 2 | 2 + 6 | 11 | 15 + 0 | 3 + 4 + 4 | 47 |
| [42] Ferry et al | 1 | 3 + 6 | 11 | 13 + 0 | 3 + 3 + 0 | 40 |
| [67] Guido et al | 2 | 4 + 7 | 9 | 15 + 3 | 3 + 2 + 0 | 45 |
| [41] Zorlu et al | 2 | 5 + 14 | 9 | 15 + 3 | 3 + 3 + 0 | 54 |
| [63] Bhide et al | 1 | 2 + 4 | 8 | 13 + 0 | 1 + 2 + 0 | 31 |
| [71] Polena et al | 2 | 4 + 6 | 11 | 15 + 10 | 4 + 3 + 0 | 55 |
| [79] Van den Bosch et al | 2 | 5 + 6 | 9 | 13 + 12 | 6 + 3 + 5 | 61 |
| [86] Ahonkallio et al | 2 | 5 + 3 | 11 | 15 + 15 | 6 + 2 + 0 | 59 |
| [53] Rezk et al | 2 | 8 + 17 | 9 | 15 + 12 | 6 + 5 + 5 | 79 |
| [52] Abdelazim et al | 2 | 5 + 14 | 9 | 20 + 2 | 3 + 1 + 5 | 61 |
| [64] Masood et al | 1 | 5 + 3 | 9 | 13 + 0 | 3 + 3 + 0 | 37 |
| [39] Seamark | 1 | 5 + 3 | 11 | 11 + 0 | 3 + 4 + 5 | 43 |
| [70] Seto et al | 2 | 5 + 11 | 9 | 20 + 9 | 6 + 5 + 5 | 72 |
| [65] Piatek et al | 2 | 3 + 3 | 9 | 13 + 10 | 6 + 4 + 6 | 56 |
| [72] Shams et al | 2 | 4 + 3 | 9 | 13 + 0 | 3 + 1 + 0 | 35 |
| Total |  |  |  |  |  | 52.8 |

***Maximum score for studies (100%): study question (2%), study population (8%), comparability of subjects (22%), Exposure or Intervention (11%), Outcome measures (20%), Statistical Analysis (19%), Results (8%), Discussion (5%), Disclosure of fundings/ Sponsorship (5%)**
